# Rapid Detection of *Legionella pneumophila* in Drinking Water, Based on Filter Immunoassay and Chronoamperometric Measurement

**DOI:** 10.3390/bios10090102

**Published:** 2020-08-20

**Authors:** Josune J. Ezenarro, Noemí Párraga-Niño, Miquel Sabrià, Fancisco Javier Del Campo, Francesc-Xavier Muñoz-Pascual, Jordi Mas, Naroa Uria

**Affiliations:** 1 Departament de Genètica i Microbiologia, Universitat Autònoma de Barcelona, E-08193 Cerdanyola, Spain; Jordi.Mas@uab.cat; 2 Waterologies S.L, C/Dinamarca, 3 (nave 9), Polígon Industrial Les Comes, E-08700c Igualada, Spain; 3 Unitat de Malalties Infeccioses, Fundació Institut de Investigació Germans Trias I Pujol, E-08916 Badalona, Spain; nparraga@igtp.cat (N.P.-N.); msabria.germanstrias@gencat.cat (M.S.); 4 Institut de Microelectrònica de Barcelona, CNM-CSIC, Esfera UAB-CEI, Campus Nord UAB, E-08193 Bellaterra, Spain; javier.delcampo@csic.es (F.J.D.C.); francescxavier.munoz@imb-cnm.csic.es (F.-X.M.-P.); 5 IKERBASQUE, Basque Foundation for Science, E-48013 Bilbao, Spain

**Keywords:** *Legionella pneumophilla*, preconcentration, immunodetection, amperometry

## Abstract

*Legionella* is a pathogenic bacterium, ubiquitous in freshwater environments and able to colonise man-made water systems from which it can be transmitted to humans during outbreaks. The prevention of such outbreaks requires a fast, low cost, automated and often portable detection system. In this work, we present a combination of sample concentration, immunoassay detection, and measurement by chronoamperometry. A nitrocellulose microfiltration membrane is used as support for both the water sample concentration and the *Legionella* immunodetection. The horseradish peroxidase enzymatic label of the antibodies permits using the redox substrate 3,3′,5,5′-Tetramethylbenzidine to generate current changes proportional to the bacterial concentration present in drinking water. Carbon screen-printed electrodes are employed in the chronoamperometric measurements. Our system reduces the detection time: from the 10 days required by the conventional culture-based methods, to 2–3 h, which could be crucial to avoid outbreaks. Additionally, the system shows a linear response (R^2^ value of 0.99), being able to detect a range of *Legionella* concentrations between 10^1^ and 10^4^ cfu·mL^−1^ with a detection limit (LoD) of 4 cfu·mL^−1^.

## 1. Introduction

*Legionella pneumophilla* is a waterborne pathogen, able to generate outbreaks that can vary in severity from non-pneumonic Pontiac fever (2–5 days illness) to Legionnaires’ disease (LD), for which the fatality rate ranges from 5 to 30% or even higher for the elderly, children, and immunosuppressed persons [1,2,3,4,5]. *Legionella pneumophila*, a ubiquitous bacterium present in many freshwater environments, is able to colonise man-made water systems such as showers, cooling towers, or whirlpool spas where it can grow unchecked and be transmitted to humans by inhalation or micro-aspiration of aerosols [6,7,8].

A good surveillance program is required to prevent such events in facilities at risk [9,10]. Currently, the gold standard methods used for *Legionella* detection are based on culture techniques (ISO 11731), which are labour-intensive, time-consuming (10 days), and require laboratory facilities [11,12]. Faster molecular methods such as PCR (polymerase chain reaction) have been developed, but these need highly skilled personnel, specific instrumentation, and are more costly [13]. Out of the 16 serogroups of *Legionella pneumophila*, serogroup 1 is responsible for the majority of the European and American isolates [14]. Some commercial kits have been developed for fast *Legionella* detection. LegionellaFast, from *Legionella* Control International, detects the presence of *Legionella pneumophila* serogroup 1 using an LFICA (Lateral Flow Immunochromatographic Assay). This device gives results within 25 min on-site without the need for special equipment or specialist expertise. However, this system is only able to detect *Legionella* serogroup 1 giving a yes/no result [15]. Legipid^®^ *Legionella* (Biotica, Castellon de la Plana, Spain) Fast Detection from Biotica is a test that combines sample concentration by filtration and magnetic immunocapture with an enzyme-immunoassay (CEIA) for the colorimetric detection of *Legionella* in water with a low limit of detection of 40 cfu. Nevertheless, the assay is carried out manually and needs to be performed by qualified personnel [16].

Over the past 20 years, biosensors have emerged as an attractive alternative for pathogen detection, since they are easy to miniaturise and automate while providing faster analysis times. Biosensors use biological recognition mechanisms to provide measurable quantitative or semi-quantitative information. A biorecognition element (e.g., enzyme, antibodies, nucleic acids, aptamer, cell receptors, and phages) binds the target of interest and a transducer (optical, electrochemical, mass-based, thermometrical or micromechanical) converts this event into a measurable signal [17,18,19]. Nevertheless, the vast majority of the biosensors developed for *Legionella* and waterborne bacteria detection still have high detection limits.

Waterborne pathogens may travel diluted in low but clinically concerning concentrations. Thus, waterborne pathogens are usually present in meagre quantities in large sample volumes. However, whereas European Commission directives state that at least 100 mL of water sample must be analysed to assess the microbiological quality of water [20,21,22], sensors are small and work with tiny sample volumes (≤1 mL). Consecuently, to include sample preconcentration steps prior to analysis is necessary [23]. Different preconcentration methods based on immunomagnetic separation (IMS) systems [24,25,26,27], centrifugation [28,29] and membrane filtration have been coupled to sensing methods. Nevertheless, membrane filtration is considered the first choice for large sample volumes (≥10 mL) [30,31].

**Figure 1:**
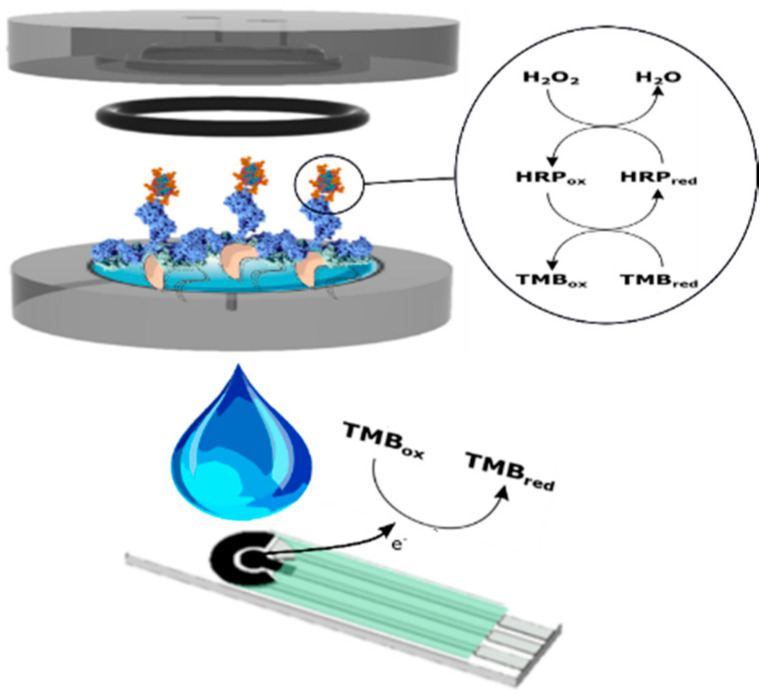
General scheme. The membrane retains the *Legionella* cells for the subsequent immunoassay and the chronoamperometric transduction of the signal.

Herein, a *Legionella pneumophila* detection system, in which a nitrocellulose microfiltration membrane acts as the support for the sample concentration as well as for the antigen-antibody reaction is proposed (Figure 1). The anti-*Legionella* antibody employed for the biorecognition is labeled with horseradish peroxidase (HRP) enzyme, which with the help of mediators such as 3,3′,5,5′-Tetramethylbenzidine (TMB), makes it possible to observe current changes proportional to the concentration of target in the samples [32]. The system can detect *Legionella* concentrations on the rage of 10^1^–10^4^ cfu·mL^−1^ with a low limit of detection (LoD) of 4 cfu·mL^−1^. Additionally, the whole process of concentration, immunoassay and chonoamperometric measurement takes only 2 h and could be integrated on the holder used for concentration.

## 2. Experimental Section

### 2.1. Microorganisms and Growth Conditions

*Legionella pneumophila* Sg1 isolated from environmental water samples was used model bacteria, as this serogroup is responsible for the majority of LD ourbreaks. Isolates were grown on buffered charcoal yeast extract culture plates (BCYE, Oxoid; Thermo Fisher Scientific, Waltham, MA, USA) for 4 days at 37 °C. Colonies from grown plates were scraped and resuspended in drinking water. Bacterial suspensions were standardised to an OD_625nm_ of 0.3 and further diluted to provide samples with a final concentration of 10^1^ to 10^5^ cfu·mL^−1^.

### 2.2. Electrode Fabrication and Electrochemical Characterisation

Chip layout was designed using Vectorworks 2016 (Techlimits, ES, Madrid, Spain) and the electrodes were screen-printed using a home-made manual press, using 25 × 25 cm/20 × 20 cm (outer dimensions/inner dimensions) screens meshed at 90 threads/cm, and using shore 75 square polyurethane squeegees. The snap-off distance was 0.5 mm for conducting inks, and 1 mm for the dielectric coating. Carbon paste C2030519P4 (Gwent Electronics Materials Ltd., Pontypool, UK) was used for printing the working and auxiliary electrodes. Silver paste Electrodag 725A (Henkel, Tetrachim, Noisiel, France) was used to print the pseudo-reference electrodes, tracks, and contact pads. A layer of UV curable dielectric Electrodag PF-455B (Henkel) was used to protect the conducting tracks between the contact pads and the electrodes and define the electrode area. These electrodes were screen printed directly on a 0.5-mm thick polyethylene terephthalate (PET) substrate (Autostat, MacDermid, Wantage, UK).

This design consisted of a central 2.5-mm diameter working electrode graphite disc surrounded by a graphite auxiliary electrode and a silver pseudo-reference electrode.

Electrochemical measurements were performed to characterise the electrode behaviour and reproducibility. These measurements were carried out with a Palm Sens4 potentiostat (PalmSens BV, Houten, The Netherlands) controlled by a PC running PSTrace 5.4 software. As redox substrate, a ready to use commercial preparation of 3,3′,5,5′-Tetramethylbenzidine (TMB, Sigma-Aldrich, Co., St Louis, MO, USA) containing hydrogen peroxide (H_2_O_2_) was used (composition not provided by the supplier). Six different electrodes were used to test the reactivity of the TMB at the electrode and analyse the reproducibility of the response. To this end, 100 µL of TMB was deposited on the electrodes and cyclic voltammetry was carried out at potentials between −200 and +600 mV at a scan rate of 50 mV. Averages and standard deviations of the current and potentials of the oxidation-reduction peaks were calculated. Similarly, to see the effect of the presence of HRP, a cyclic voltammetry was performed after depositing a mixture of 50 µL of TMB with 50 µL of antibody (0.2 ng·mL^−1^).

### 2.3. Legionella Pneumophila Concentration and Antibody Reaction

A concentration and detection protocol optimised and reported by our research group in a previous work was followed for the *Legionella pneumophila* detection [14]. Employing a custom-made holder, different inoculum between 10^1^ and 10^5^ cfu·mL^−1^ in a final volume of 200 mL of drinking water were filtered through a 25-mm diameter nitrocellulose (NC) membrane (Whatman Nitrocellulose, GE Healthcare Life Science, Buckinghamshire, UK) with a nominal pore of 0.2 µm at a flow rate of 0.5 mL·s^−1^ by a peristaltic pump (XX8000230, Merck Millipore, Burlington, VT, USA). Similarly, 200 mL of drinking water without bacterial cells was filtered as blank. After filtration, membranes were transferred to a 6-well plate and air-dried for 5 min to carry out the different steps of the on-filter immunoassay. Next, membranes were incubated in 2 mL of 1% *v*/*v* Tween-20 (Sigma-Aldrich) in 0.01 M phosphate-buffered saline (PBS, Sigma-Aldrich) for 30 min. After that, the samples were treated with 2 mL of LP3IIG2 anti-*Legionella* antibody-HRP at a final concentration of 0.5 µg·µL^−1^ in PBS for 1 h. After this, the membranes were washed three times with 2 mL of 0.5% Tween-20 in PBS and once with only PBS solution for 5 min. Subsequently, the membranes were transferred to a new 6-well plate and 150 µL of TMB was added. A reaction time of 16 min between the HRP enzyme and TMB substrate was defined. In previous experiments, it was observed that a 16-min period was sufficient to obtain a stable signal.

The antibody was selected from a previous work [33] as a specific sensitive antibody for the detection of *L. pneumophila*. This study confirmed that the LP3IIG2 antibody was able to recognise almost all the serogroups of *L. pneumophila* and did not cross-react with other microbial species.

The holder employed to perform the filtrations at this study is described more in detail in previous work [34].

### 2.4. Electrochemical Measurements for Legionella Detection

Following the 16-min immunodetection reaction, 100 µL of the reacted TMB was deposited on the electrode, and the current was measured at 50 mV vs. Ag (determined from the cyclic voltammetry performed to characterise the electrodes) for 240 s (time enough to stabilise the current signal). Finally, we compared the current values at different times during the chronoamperometry (10, 25, 50, 100 and 240 s) for the different *Legionella* concentrations. All measurements were carried out in triplicate, and the averages and standard errors were calculated. The limit of detection of the system was calculated as the bacterial concentration equal or higher to the current signal value calculated as the blank current plus 3 times the blank standard deviation.

## 3. Results and Discussion

### 3.1. Electrode and Redox Substrate Characterisation

**Figure 2:**
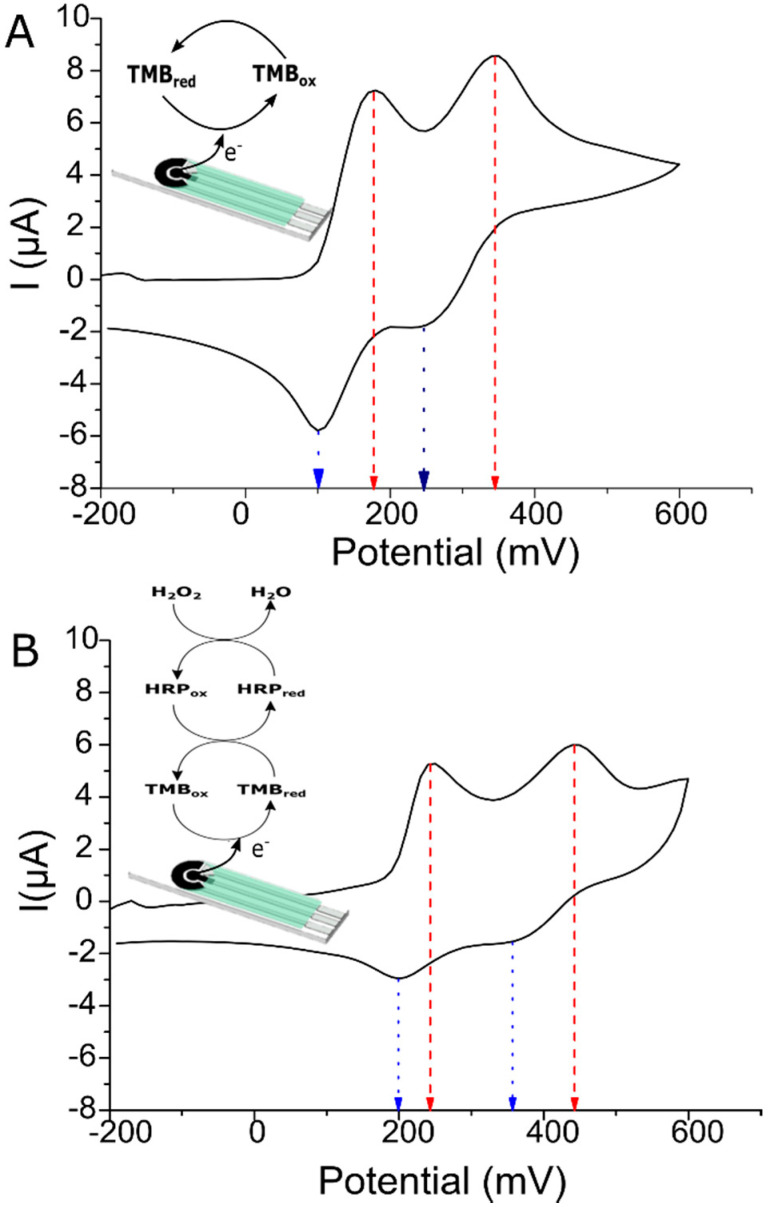
(**A**) Cyclic voltammetry of the substrate 3,3′,5,5′-Tetramethylbenzidine (TMB) in a screen-printed electrode (SPE). (**B**) Cyclic voltammetry of the substrate 3,3′,5,5′-Tetramethylbenzidine (TMB) and horseradish peroxidase (HRP) reaction. Peak potentials are highlighted in red (oxidation) and blue (reduction).

The fabricated screen-printed electrodes were analysed by cyclic voltammetry (CV) using TMB, the chromogenic and redox substrate employed later in the *Legionella* detection protocol (Figure 2). TMB is one of the most used peroxidase substrates, as its oxidation mechanism is well-known [35], and it offers the possibility of performing either optical or electrochemical measurements.

TMB undergoes a two-electron oxidation-reduction process [35,36]. We confirmed this by cyclic voltammetry using our electrodes (Figure 2A). Two oxidation peaks at 180 mV and 350 mV (vs. Ag) (red dashed arrows) and two reduction peaks at 250 mV and 100 mV (vs. Ag) (blue dotted arrows), respectively, were observed. Moreover, CVs were performed using six different screen-printed electrodes (SPE) to test the reproducibility. Good reproducibility of the electrodes was observed with variabilities of 3% and 7% for the current intensity and the potential of the peaks, respectively.

TMB reaction with anti-*Legionella* antibody labeled with HRP enzyme was also characterised by our electrodes. When HRP was involved in the redox reaction of TMB (Figure 2B), the CV continued showing two oxidation (250 mV and 440 mV vs. Ag) and two reduction peaks (350 mV and 200 mV vs. Ag). However, compared to the CV observed when HRP is not involved in the reaction (Figure 2A), the peak potentials shifted to higher potentials and the measured peak currents were lower. This is attributed to the action of the H_2_O_2_ present in the enzymatic substrate solution, which can oxidise the Ag pseudo-reference electrode but also passivate the working electrode, particularly under our pH conditions. The Figure 2B inset demonstrates that the HRP enzyme oxidises the TMB substrate and it is reduced back at the electrode surface. Thus, higher enzyme concentrations and activities lead to higher reduction currents. Based on the voltammetric responses shown in Figure 2A,B, a working potential of 50 mV vs. Ag was selected. Besides, the current background at the selected potential is near zero, which prevents interferences from the direct oxidation of the substrate H_2_O_2_ or the reduction of dissolved oxygen. These conditions are suitable for measuring low amounts of product in the presence of high substrate concentrations [37,38].

In case of the measurement of an ideal blank (without *Legionella*), no HRP-labelled antibody should be present in the electrode and CV very similar to the one depicted in Figure 2A should be observed, confirming the suitability of a polarisation potential of 50 mV (vs. Ag).

### 3.2. Calibration Curve for the Detection and Quantification of Legionella

**Figure 3:**
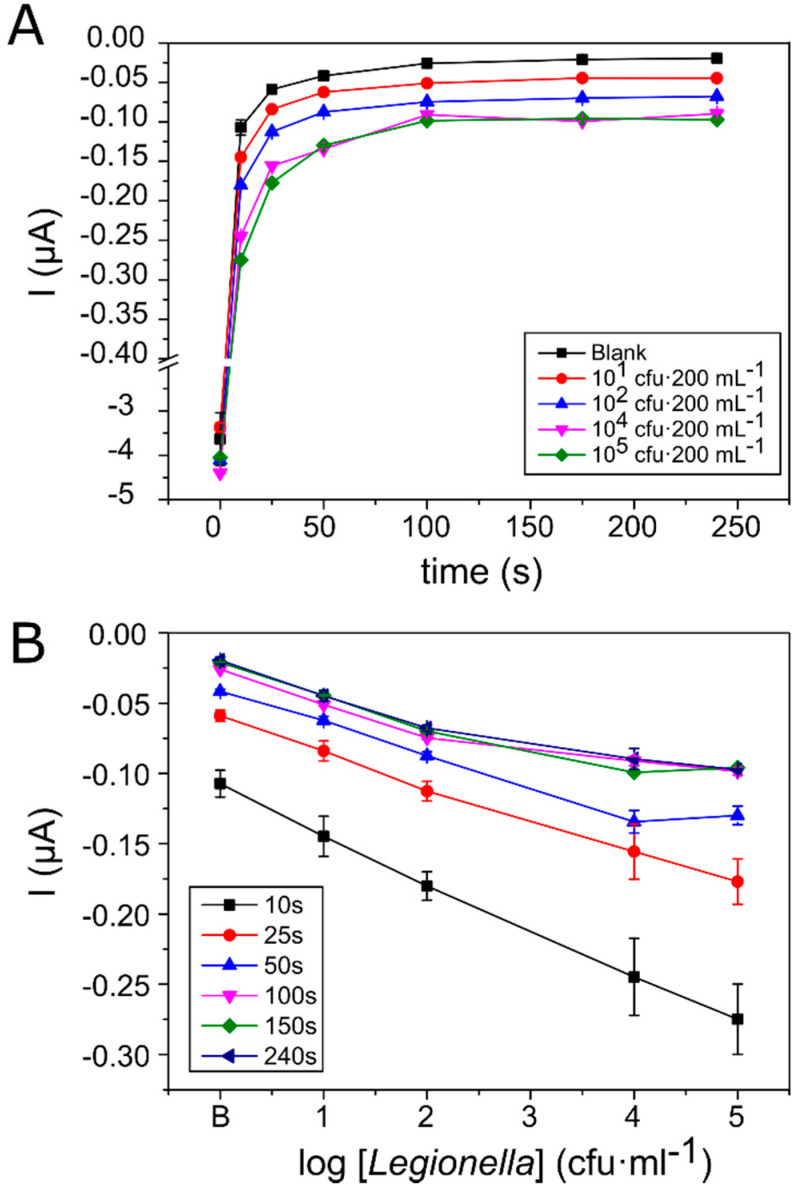
(**A**) Current change response in time, measured for different *Legionella* concentrations. (**B**) Current changes measured for different *Legionella* concentrations at different times (from 0 to 240 s). Standard error bars correspond to the measurements made in three different culture replicates of each concentration measured in two different assays (*n* = 6).

To test the capacity of the system to detect *Legionella pneumophila* in water, 200 mL of drinking water containing different *Legionella* concentrations ranging between 10^1^ and 10^5^ cfu·mL^−1^ was filtered. Then, once immunoassay and incubation with the TMB substrate were carried out, chronoamperometric measurements were carried out at a reduction potential of +50 mV vs. Ag for a total of 240 s (Figure 3).

Figure 3A shows that after a fast current decrease during the first 50 s resulting from electrode polarisation, the reaction at the electrode stabilised and started to reach a plateau state. Additionally, it was observed that differences in the current values obtained by the different *Legionella* concentrations were lower, particularly among 10^4^ and 10^5^ cfu·mL^−1^, the highest concentrations analysed. This is clearly observed in Figure 3B, where current values were represented in relation to *Legionella* concentration at different reaction times. The results show a linear relationship between *Legionella* concentration and the current obtained in short measurement periods between 10 and 25 s. However, after 50 s of reaction, linearity was lost at the highest concentration of *Legionella*, probably as a consequence of the passivation of the electrodes related to TMB precipitation. When HRP oxidises TMB, the resultant blue product deposits on the electrode surface, blocking it and reducing the current obtained [39,40]. At high bacterial concentrations, TMB is oxidised faster and therefore, the passivation effect is observed sooner. In addition to linearity loss, this could also explain the large standard deviations found at high bacterial concentrations.

Finally, despite the loss of linearity at the highest bacterial concentration (10^5^ cfu·mL^−1^), we decided to use the readings taken after 50 s because they provided an equilibrium between good dynamic range (10^1^ to 10^4^ cfu·mL^−1^) and low variability.

**Figure 4:**
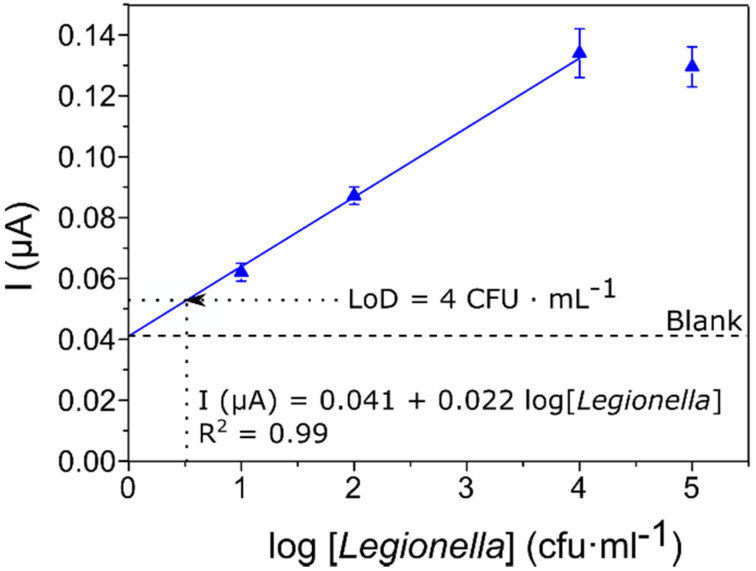
Sensor calibration curve, where the current obtained at a constant potential of 50 mV after 50 s of the chronoamperometry is expressed in absolute values as a function of the logarithm of the increasing concentrations of *Legionella* from 0 (blank) to 10^5^ cfu·mL^−1^. The regression line is indicated in blue and error bars represent the standard error (*n* = 6).

In Figure 4, the absolute values of stable current recorded at time 50 s have been plotted as a function of the concentration of *Legionella pneumophila* present in the samples.

A regression line was fitted to the values (R^2^ = 0.99), excluding the last point at which TMB precipitation on top of the electrodes caused passivation and unreliable readings. The resolution of the curve fitting indicated a sensitivity (expressed as the slope of the I vs. log[*Legionella*] curve) of 22 nA/log[*Legionella*] in the range of 10^1^ to 10^4^ cfu·mL^−1^.

Additionally, the detection limit of our system was determined taking into account the variability of the chronoamperometric current obtained in blank samples (samples without *Legionella*). This limit was established as a current value of 0.053 µA corresponding to a *Legionella* concentration of about four cells per milliliter. We conclude that the proposed method can detect the presence of *Legionella* at low concentrations but without any of the disadvantages of current standard methods.

Currently, ISO 11731:2017 and ISO/TS 12869:2019 are by far the most used methods for isolation and estimation of *Legionella* in drinking water. On the one hand, ISO 11731:2017 is a culture-based method where a concentration by membrane filtration is needed for the detection of less than 10^4^ cfu·L^−1^ [41]. Nevertheless, despite its high sensitivity and ability to comply with the 10^2^–10^3^ cfu·L^−1^ standard set by most regulatory agencies [42,43], it requires highly skilled personnel and takes 7–10 days to provide results. On the other hand, ISO/TS 12869:2019, which is based on the quantitative polymerase chain reaction (qPCR) [44], is faster and simpler than ISO 11731:2017 and provides similar sensitivity. However, it needs sophisticated instrumentation and qualified personnel.

Methods based on biosensors are gaining attention due to the advantages they offer in terms of miniaturisation and automation, providing fast and user-friendly detection devices. Notwithstanding, currently available biosensors lag behind in terms of sensitivity and in most cases are unable to match therequirements of the current regulatory frameworks [45]. For this reason, one of the requirements for on-site detection devices is to integrate the capacity to preconcentrate (cells) or amplify (nucleic acids) the target [46].

Genosensors detect DNA-, RNA- or PCR-amplified products that come from the target cells. However, nucleic acid extraction and concentration are difficult to automate. Although some portable devices based on µPCR [47] on-chip or loop-mediated isotermal amplification (LAMP) [48] exist for nucleic acid amplification, they are mostly laboratory techniques that require expensive equipment and qualified personnel. In addition, these techniques are restricted to small sample volumes [49,50,51]. Moreover, most of these methods express their detection limits as concentration of nucleic acids, without stating clearly the equivalence to actual *Legionella* concentrations. Thus, although quantitative methods detecting nucleic acids, such as the ISO 12869:2019 exist, these biosensors provide qualitative results. [52,53,54,55,56,57]. Additionally, the biggest weakness of genosensors is their inability to detect viable but not culturable (VBNC) microorganisms [58]

Immunosensors for *Legionella* are generally based on sandwich immunoassays in which a first antibody immobilised on the sensor captures the target, while a secondary labelled antibody transduces the bio-recognition into a measurable signal. Nevertheless, the use of a two-antibody system makes the analysis more expensive. Moreover, they have high detection limits in the range of 10^6^–10^8^ cfu·L^−1^ [30,59,60,61,62] and, the one able to achieve a lower detection limit (10^4^ cfu·L^−1^) needed a preconcentration step and even so, it did not reach the standards established for *Legionella* surveillance [30].

Membrane-based methods seem to be currently the only methods capable of handling volumes from millilitres to litres. However, their integration in detection systems remains challenging [31]. The system we present in this work integrates concentration and immunoassay by the use of a microfiltration membrane as a support for both. Thus, the sample concentration process is already included in the detection protocol allowing adherence to the analysis sample volume requirements established by regulations. To carry out the whole process (concentration, as well as the different steps of the immunoassay and the chronoamperometric measurement) takes 2–3 h to provide reliable results. This may seem long compared to the 30 to 45 min reported by some genosensors [53,54,55], but these claims do not take into account the time needed for sample concentration preparation. Additionally, as cells are retained in the membrane due to the filtration step, there is no need for a capture antibody. Thus, a single antibody system is used for the immunoassay which lowers the cost for each test.

## 4. Conclusions

Effective *Legionella* monitoring and surveillance in drinking water systems is essential in order to avoid outbreak appearance. In this work, we have developed an electrochemical immunoassay system that provides the advantage of utilising a microfiltration membrane that acts as the support for sample concentration and immunodetection, giving the chance to treat large sample volumes and increase the possibility to detect low *Legionella* amounts with a detection limit of 4 cfu·mL^−1^.

Additionally, both concentration and immunodetection steps could be integrated into a single holder in the near future, allowing us to obtain a more straightforward and faster system that gives results within 2 h. Thus, the developed detection system is able to overcome one of the most significant drawbacks of the gold standard method ISO 11731, the detection time.

As a result, we have accomplished the objective of obtaining a rapid, economical and user-friendly system for *Legionella pneumophila* detection. Moreover, the system was specially designed in such a way that in future versions all steps can be automated and carried out by micropumps without the need for qualified personnel and fabricated with low-cost materials that could easily be mass-produced.
